# Red–Blue and White Supplementary Light Increase Carotenoid Accumulation and Improve Fruit Quality in Satsuma Mandarin: Associations with Chlorophyll Content and Carotenogenic Gene Expression

**DOI:** 10.3390/plants15193067

**Published:** 2026-10-08

**Authors:** Wei Hu, Wenli Yu, Dechun Liu, Zhonghua Xiong, Li Yang, Liuqing Kuang, Jie Song, Jingheng Xie, Yong Liu

**Affiliations:** College of Agronomy, Jiangxi Agricultural University, Nanchang 330045, China; weihu@jxau.edu.cn (W.H.); ywl6756@163.com (W.Y.); ldc873380800@163.com (D.L.); xzhchh@163.com (Z.X.); yangli526526@126.com (L.Y.); kuangliuqing@jxau.edu.cn (L.K.); songjie507@163.com (J.S.); xiejingheng@126.com (J.X.)

**Keywords:** *Citrus unshiu*, supplementary lighting, carotenoid biosynthesis, fruit nutritional quality, gene expression regulation

## Abstract

Light regulates plant growth and secondary metabolism. To improve the nutritional quality and economic benefits of satsuma mandarin (*Citrus unshiu* Marc.) under greenhouse conditions with reduced natural light, we investigated the effects of supplementary lighting with different light qualities and intensities on fruit coloration, carotenoid accumulation, and fruit quality. Supplementary lighting was applied from 156 days after flowering using red, blue, red–blue combinations, and white light. All treatments significantly increased the color contribution index and total carotenoid content in both the rind and flesh compared with the control, with the red–blue light RB14 and white light W1 treatments being the most effective. β-Cryptoxanthin, the major carotenoid in satsuma mandarin, increased by 120.3% and 97.6% under RB14 and W1, respectively. Supplementary lighting also significantly increased the soluble sugar, total soluble solid, ascorbic acid, amino acid, flavonoid, and phenolic acid contents at most sampling time points. The leaf chlorophyll content increased, and chlorophyll degradation was delayed. qRT-PCR showed that RB14 and W1 upregulated the key carotenoid biosynthesis genes *CitPSY*, *CitPDS*, *CitZDS*, *CitCRTISO*, *CitLCYE*, *CitLCYB*, *CitBCH2*, *CitZEP*, and *CitVDE* and downregulated *CitCCD4*. These results suggest that supplementary lighting may enhance carotenoid accumulation and improve fruit quality by increasing the chlorophyll content and modulating carotenogenic gene expression, thereby providing a theoretical basis for citrus quality improvement.

## 1. Introduction

China is currently the world’s largest citrus producer [1]. In 2024, total citrus production in China reached 67.915 million tons, with a per capita availability of 48 kg [2], far exceeding the world average of 21 kg. This has resulted in a structural surplus in the citrus market and a marked decline in the comparative profitability of citrus production. Consumer demand has long shifted from “eating enough” to “eating well”; accordingly, citrus production must shift from “increasing yield” to “improving quality and efficiency.” Carotenoids are the main pigments responsible for citrus coloration and are also nutrients with important health benefits. Increasing the carotenoid content in citrus fruits not only improves fruit coloration and consumer appeal but also greatly enhances nutritional value, because carotenoids exhibit high antioxidant activity in the human body, can delay aging and protect against various diseases, and are precursors of vitamin A [3,4].

Carotenoid accumulation in citrus fruit is influenced by multiple factors, including cultivar and environmental factors such as temperature and light. Variation in the carotenoid composition and content among citrus cultivars, such as sweet orange, satsuma mandarin, grapefruit, and lime, leads to marked differences in rind and flesh color [5]. In general, low temperature favors carotenoid accumulation and promotes fruit coloration during citrus fruit ripening, whereas high temperature delays coloration [6,7]. For example, low temperature promotes rind degreening and color development in satsuma mandarin [8], and Newhall navel orange grown in cooler regions also shows better coloration [7]. Light is a key regulator of plant growth and development and is the energy source for photosynthesis; it acts through different light intensities, light qualities, and photoperiods [9]. In addition, light is a key signal mediating carotenoid accumulation in horticultural plants [5]. It has been observed that fruit developing on the outer canopy and directly exposed to sunlight develops a brighter orange color than shaded fruit inside the canopy [10]. Shading of citrus fruit reduces carotenoid accumulation and downregulates the expression of carotenoid biosynthesis genes [11]. Carotenoids such as lycopene and β-carotene are derived from the key precursor geranylgeranyl diphosphate (GGPP), and GGPP biosynthesis uses photosynthetic products as substrates [12]; carotenoids are also important pigments involved in photosynthesis [13]. Therefore, photosynthate accumulation is closely related to carotenoid content. However, this does not mean that higher light intensity is always more conducive to pigment synthesis. Studies have shown that strong light accelerates chlorophyll degradation [14]. Thus, an appropriate light intensity can improve photosynthetic efficiency and promote pigment synthesis. Light quality also affects carotenoid metabolism, and red light promotes the accumulation of β-cryptoxanthin and total carotenoids in citrus rind by upregulating the expression of carotenoid biosynthesis-related genes [15]. Similarly, blue light treatment significantly increases the carotenoid content in the flesh of satsuma mandarin and Valencia orange by upregulating the expression of carotenoid biosynthesis-related genes [16]. Yellow LED light treatment has been reported to increase the total phenolic content and antioxidant capacity in postharvest apple and sweet pepper fruit, accelerate fruit coloration, and significantly increase the contents of β-carotene and lutein [17]. Combinations of red and blue light with other light qualities (green, orange, or yellow) are beneficial for the accumulation of sugars, β-carotene, and other nutrients in cruciferous plants [18].

In addition, light influences chlorophyll synthesis and degradation, thereby regulating photosynthate accumulation [19] and ultimately determining fruit quality. Previous studies have suggested that blue and red light are among the most effective spectral regions for photosynthesis. For example, combinations of red, blue, and white light have been shown to enhance photosynthetic capacity in tomato by promoting the expression of *SlLHCB* and *SlLHCA*; significantly increasing the contents of chlorophyll, carotenoids, and anthocyanins; and ultimately increasing tomato biomass [20]. Moreover, supplementary red and blue light has been reported to significantly improve fruit quality traits such as soluble solids, soluble sugars, and flavonols in blueberry [21]. Sun et al. [22] also reported that combined supplementary red and blue light significantly enhanced the accumulation of nutritional compounds in tomato fruit, including soluble sugars, ascorbic acid, amino acids, total phenolic acids, and lycopene.

Carotenoid metabolism is regulated by multiple genes. Since phytoene biosynthesis is the first rate-determining step in carotenoid biosynthesis, phytoene synthase (PSY) is the rate-limiting enzyme [5]. Overexpression of *PSY* in transgenic Brassica napus has been reported to increase carotenoid accumulation [23]. In addition, several studies have reported that the transcript levels of ζ-carotene desaturase (ZDS) are positively correlated with carotenoid levels in fruits such as apricot, citrus, and kiwifruit, indicating that *ZDS* is also a major regulatory step in fruit carotenoid biosynthesis [24,25,26]. Carotenoid isomerase (CRTISO) is a key enzyme that isomerizes poly-cis-carotenoids to all-trans-carotenoids [27] and plays a critical role in carotenoid synthesis. During melon ripening, almost all upstream genes in the methylerythritol phosphate (MEP) pathway for β-carotene biosynthesis are significantly upregulated, whereas the downstream genes *LCYE* and *BCH* are downregulated, together leading to the substantial accumulation of β-carotene [28]. Among citrus fruits, satsuma mandarin mainly accumulates β-cryptoxanthin, Valencia orange mainly accumulates violaxanthin, and lemon accumulates only low carotenoid levels; these differences are caused by the differential expression of carotenoid biosynthesis genes such as *PSY*, *PDS*, *ZDS*, *LCYB*, *HYB* (*BCH*), and *ZEP* [29].

Satsuma mandarin is the most widely cultivated citrus variety in China [30]. However, its concentrated harvest season, unstable fruit quality, and high incidence of physiological disorders such as fruit peel puffiness and peel creasing are limiting factors in its cultivation [31]. Therefore, protected cultivation has been adopted to extend the market supply period and improve fruit quality. However, protective cultivation facilities reduce sunlight intensity, especially in winter when daylength and solar radiation decrease. Moreover, greenhouse temperatures are higher than ambient outside temperatures, and the combination of these two factors influences carotenoid accumulation in fruit. To further improve the nutritional value and coloration of satsuma mandarin fruit, this study used artificial supplemental red–blue or white light in a protected greenhouse to extend the photoperiod. We investigated the effects of different light qualities and light intensities on fruit quality attributes, including peel and flesh color and carotenoid content; identified the optimal supplementary lighting treatment; analyzed the effects of supplementary light on the leaf chlorophyll content and expression of carotenoid biosynthesis-related genes in fruit; and preliminarily elucidated the physiological and molecular mechanisms by which supplementary light increases the carotenoid content.

## 2. Results

### 2.1. Supplementary Light Promotes Rind Coloration and Increases Carotenoid Content

**Figure 1:**
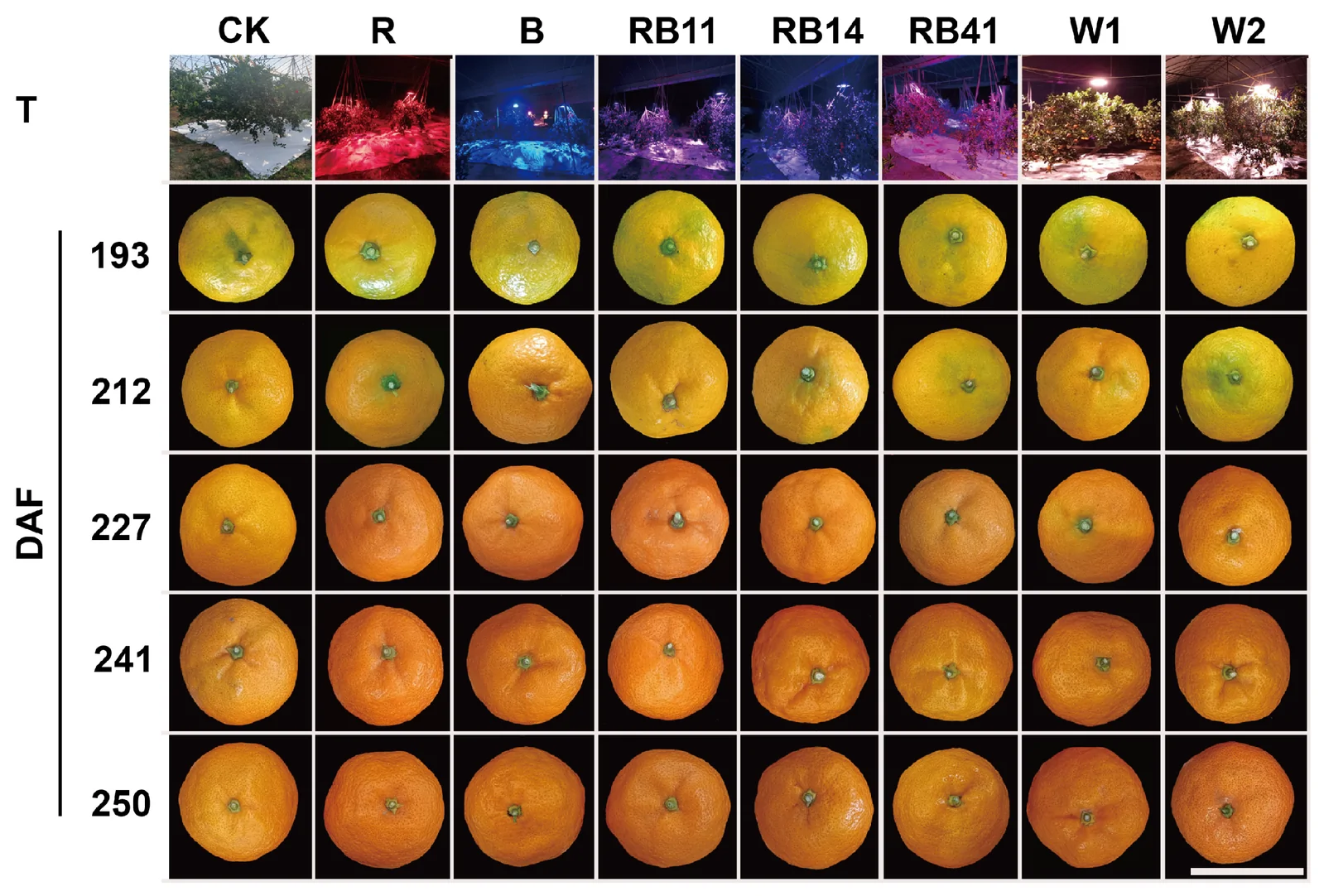
Rind coloration of satsuma mandarin fruit under different supplementary light treatments.

Supplementary lighting was initiated at 156 days after full bloom (DAF). After the onset of fruit coloration, the peel color was recorded approximately every 15 days starting from 193 DAF. From 227 DAF onward, peel coloration in all light-treated groups was visibly more intense than that in the control; thereafter, the control showed little further change, whereas the supplementary light treatments maintained markedly higher rind coloration (Figure 1).

**Figure 2:**
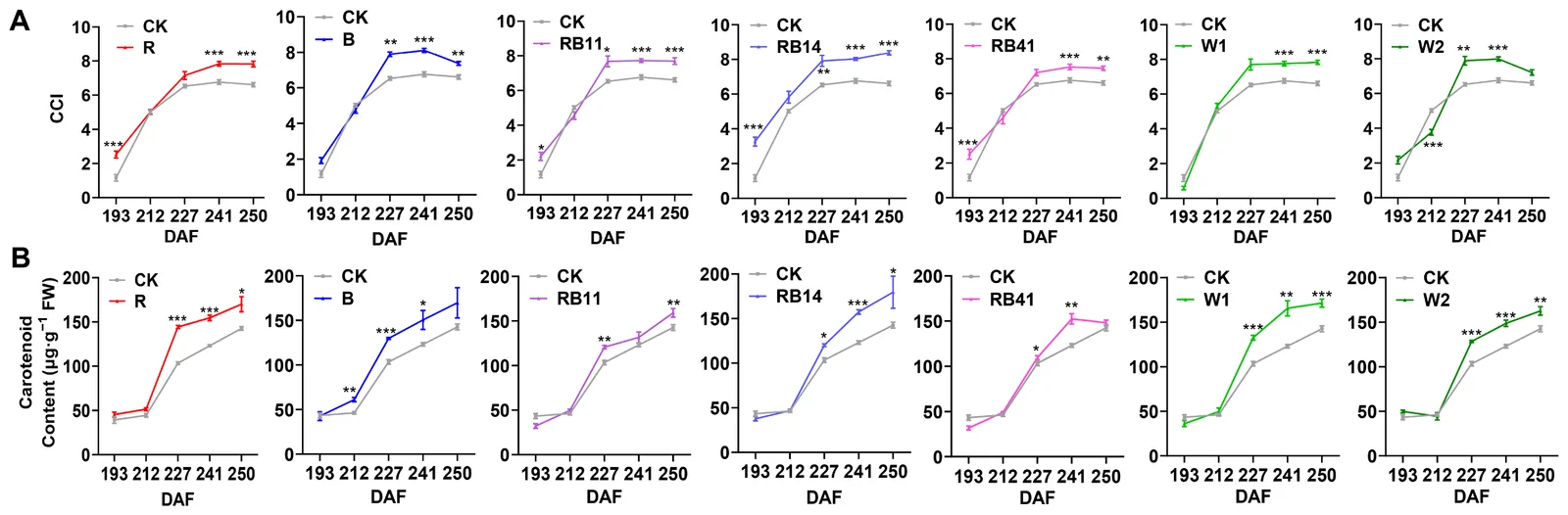
Rind color contribution index (CCI) and carotenoid content of fruit under different supplementary light treatments. (**A**) Changes in rind CCI. (**B**) Changes in rind carotenoid content. * *p* < 0.05; ** *p* < 0.01; and *** *p* < 0.001 (Dunnett’s test).

Both the control and light-treated fruits exhibited a rapid increase in the peel color index (CCI) from 193 DAF to 227 DAF, followed by smaller changes. After 227 DAF, the CCI values in all light treatments except W2 were significantly higher than those in the control. Overall, treatments R, RB14, and W1 had the greatest effect on the peel CCI (Figure 2A). The pattern of total carotenoid content in the peel was similar to that of the CCI, with a rapid increase from 193 DAF to 227 DAF, followed by a slower phase. In terms of the magnitude of carotenoid increase, treatments R, RB14, and W1 again performed best (Figure 2B). These results indicate that all supplementary light treatments significantly increased the peel carotenoid content and promoted fruit coloration, with R, RB14, and W1 being the most effective.

### 2.2. Supplementary Light Increases Flesh Carotenoid Content

**Figure 3:**
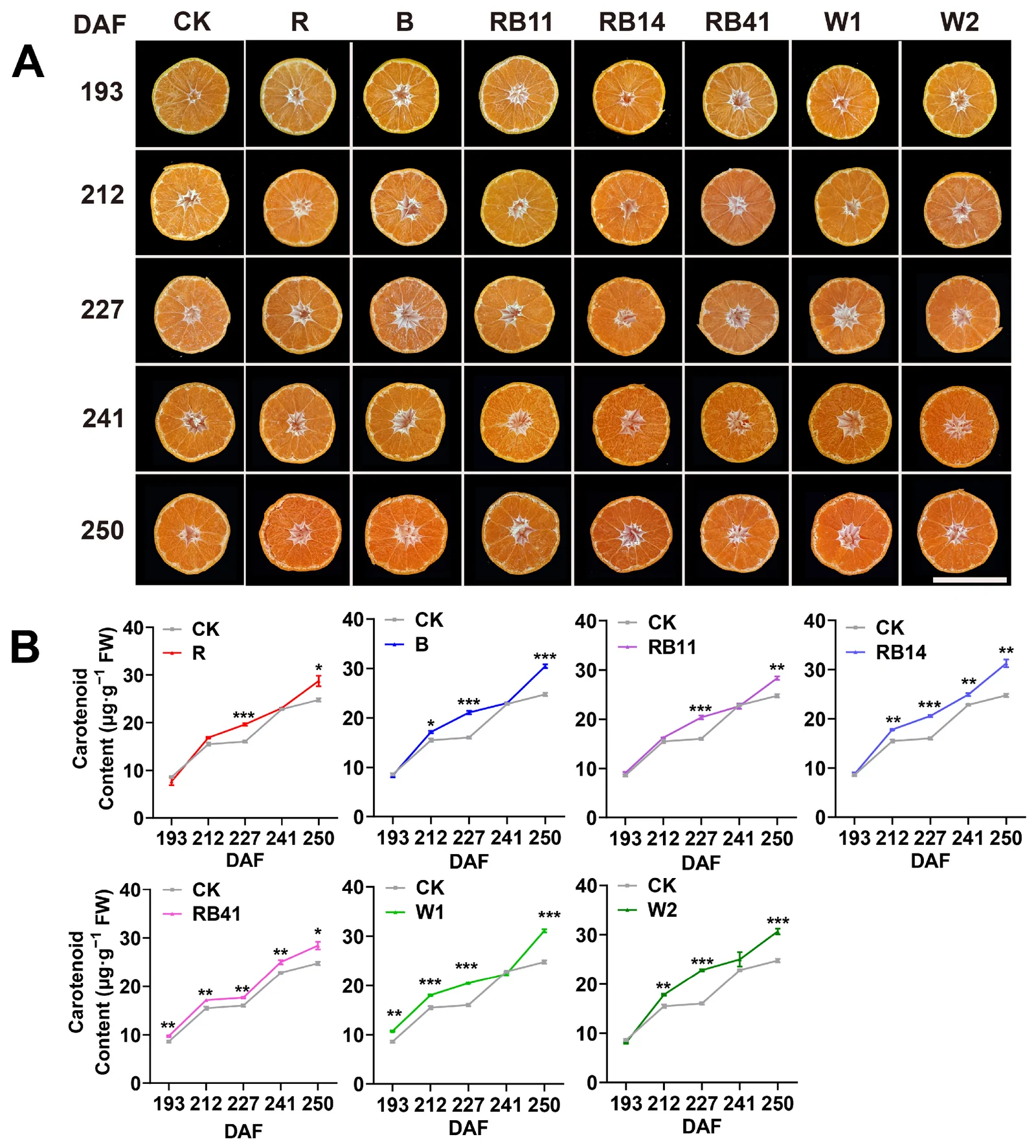
Flesh color and carotenoid content of fruit under different supplementary light treatments. (**A**) Changes in flesh color. (**B**) Changes in flesh carotenoid content. * *p* < 0.05; ** *p* < 0.01; and *** *p* < 0.001 (Dunnett’s test).

Based on fruit cross-section images, flesh color differed little from the control at 193 DAF. However, starting from 212 DAF, the flesh color in the RB14, RB41, W1, and W2 treatments was visibly redder than that in the control at various time points, and by 250 DAF, flesh color in all treatments was deeper than that in the control (Figure 3A). Unlike peel carotenoids, which increased rapidly from 193 DAF to 227 DAF and then slowed, the total flesh carotenoid content increased steadily from 193 DAF to 250 DAF at a nearly constant rate, reaching maximum values at 250 DAF in both the control and light treatments (Figure 3B). This indicates that carotenoid accumulation patterns differ between the flesh and the peel. Based on flesh color and carotenoid content, RB14, W1, and W2 were the most effective treatments.

### 2.3. Supplementary Light Improves Fruit Quality

In addition to carotenoids, other fruit nutritional quality traits were analyzed. The results showed that under the control and the R, RB11, W1, and W2 treatments, the total soluble solids (TSSs) content generally exhibited an initial increase followed by a decrease over time, whereas under B, RB14, and RB41, it generally showed an increasing trend. From 227 DAF onward, the mean TSSs content under all supplementary light treatments was higher than that of the control (Table 1), and overall, the highest mean values were recorded for RB14 and W1. The soluble sugar content showed a similar trend to TSSs, but from 212 DAF to 241 DAF, the mean soluble sugar content under all supplementary light treatments was higher than that of the control, while at 250 DAF, no significant differences were detected among treatments (Table 2). Titratable acidity generally decreased over time in the control and all treatments, and except at some time points, no significant differences were found between the light treatments and the control (Table 3). The ascorbic acid content showed a trend similar to that of TSSs, and the treatments with the highest mean values were also RB14 and W1 (Table 4).

**Figure 4:**
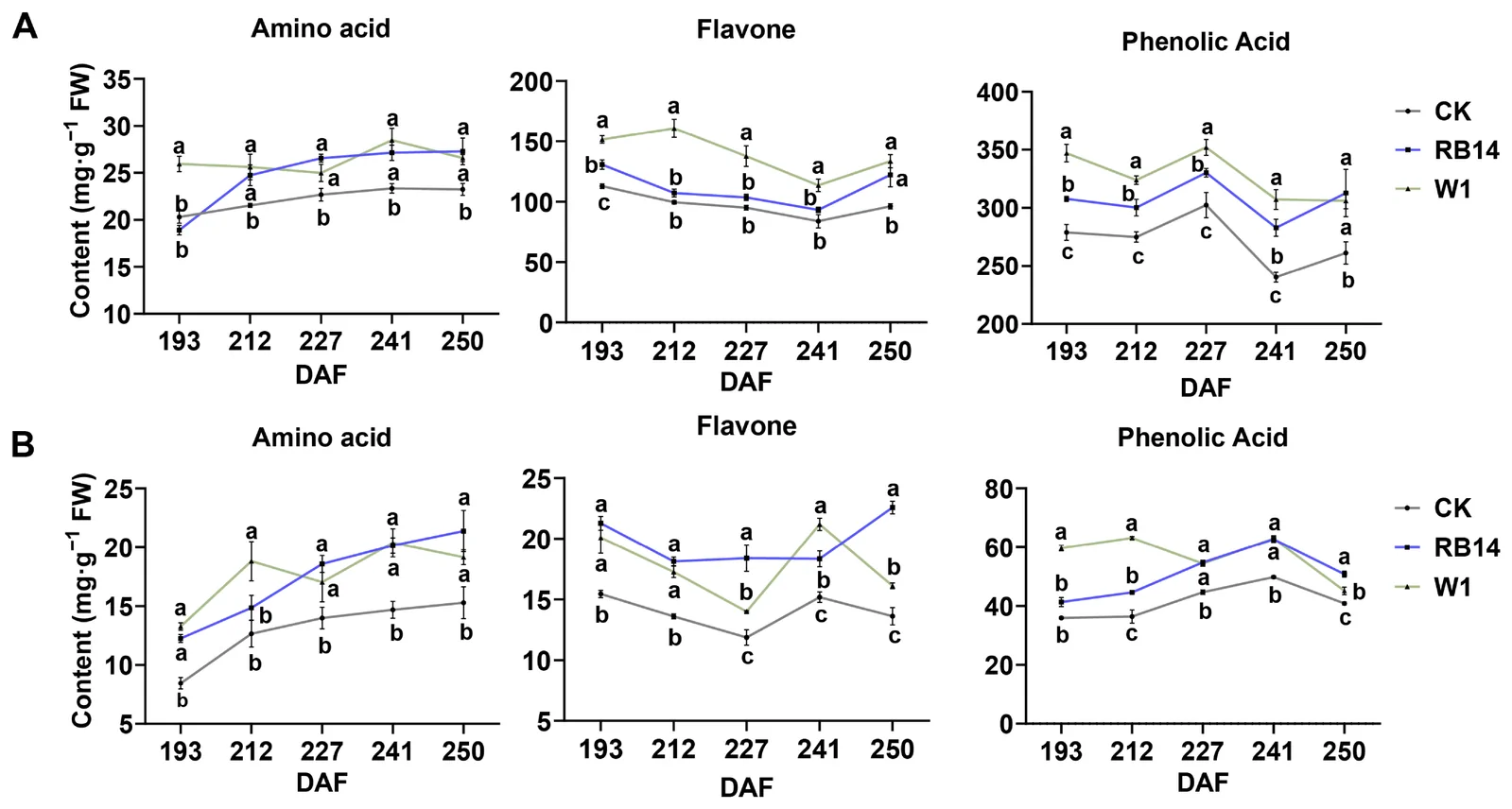
Fruit quality under different supplementary light treatments. (**A**) Changes in rind amino acid, flavone, and phenolic acid contents under RB14 and W1 treatments. (**B**) Changes in flesh amino acid, flavone, and phenolic acid contents under RB14 and W1 treatments. Different lowercase letters indicate significant differences among treatments at the same time point according to Tukey’s HSD test (*p* < 0.05).

Based on the above results for peel and flesh carotenoid contents and fruit quality, RB14 and W1 were identified as the most effective treatments; therefore, amino acid, total flavonoid, and phenolic acid contents were measured in the peel and flesh of these two treatments. In the peel, the amino acid content was significantly higher in RB14 and W1 than in the control at all time points except 193 DAF for RB14. W1 treatment significantly increased the total flavonoid and phenolic acid contents at all time points, while RB14 treatment significantly increased the phenolic acid content at all time points but only significantly increased the total flavonoid content at 193 DAF and 250 DAF (Figure 4A). In the flesh, W1 treatment significantly increased the amino acid, total flavonoid, and phenolic acid contents at all time points. RB14 treatment significantly increased the amino acid content at all time points except 212 DAF, significantly increased the total flavonoid content at all time points, and significantly increased the phenolic acid content except at 193 DAF (Figure 4B). Overall, RB14 and W1 significantly increased the contents of amino acids, total flavonoids, and phenolic acids in satsuma mandarin fruit.

### 2.4. Supplementary Light Increases Leaf Chlorophyll Content

**Figure 5:**
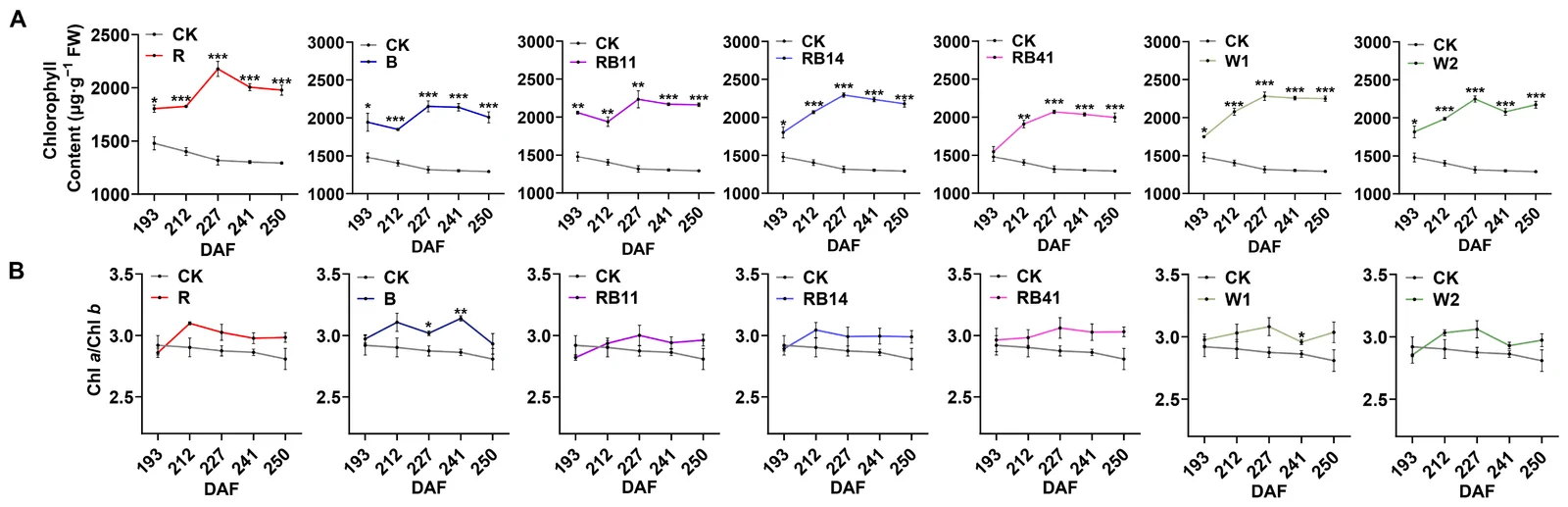
Leaf chlorophyll content and chlorophyll a/b ratio under different supplementary light treatments. (**A**) Changes in leaf chlorophyll content. (**B**) Changes in leaf chlorophyll a/b ratio. * *p* < 0.05; ** *p* < 0.01; and *** *p* < 0.001 (Dunnett’s test).

To explore the potential physiological mechanisms by which supplementary light increases fruit nutritional quality, including carotenoids, we analyzed the leaf chlorophyll content and its dynamics after supplementary lighting. In all light treatments, the leaf chlorophyll content increased before 227 DAF, peaked at 227 DAF, and then declined. Except for RB14, all light treatments had significantly higher chlorophyll contents than the control at various time points, whereas the control showed a decreasing trend in total leaf carotenoid content from 193 DAF to 250 DAF (Figure 5A). This indicates that supplementary light significantly increased the leaf chlorophyll content and delayed chlorophyll degradation. Among the treatments, RB14, W1, and W2 showed greater increases in chlorophyll content. The chlorophyll a/b ratio in the control decreased from 193 DAF to 250 DAF. Except for treatments B and W1, the chlorophyll a/b ratio in the other treatments did not differ significantly from the control, but their mean values were mostly higher than those of the control. Treatment B was significantly higher than the control at 227 DAF and 241 DAF, whereas W1 was significantly higher only at 241 DAF (Figure 5B). The actual chlorophyll a and b contents are shown in Table S1. Since the chlorophyll a/b ratio reflects leaf senescence [32], these results suggest that supplementary light delays leaf senescence.

### 2.5. Supplementary Light Increases the Contents of Carotenoid Components in Flesh

**Figure 6:**
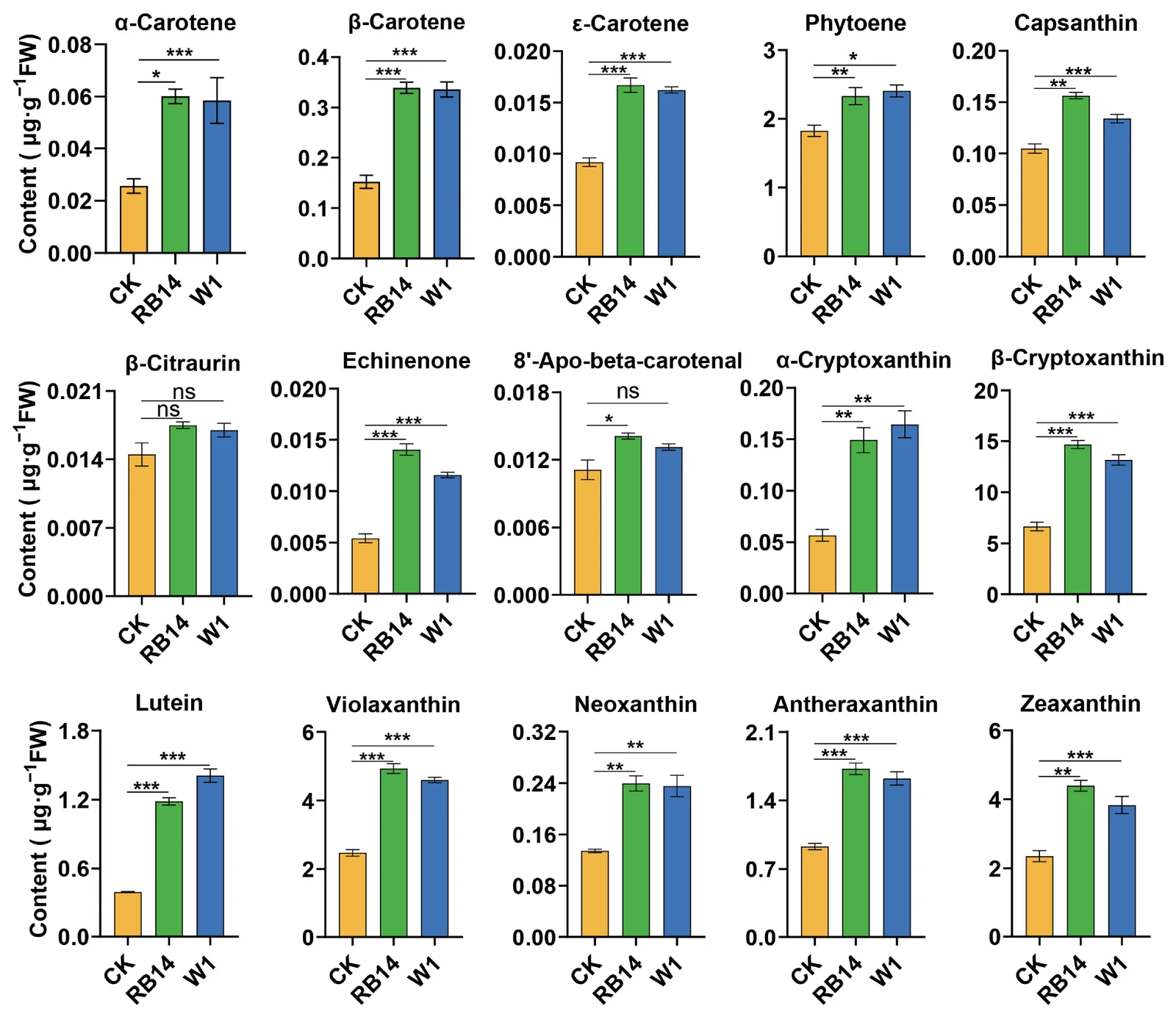
Carotenoid composition and content in the flesh under RB14 and W1 treatments. ns, no statistically significant difference; * *p* < 0.05; ** *p* < 0.01; and *** *p* < 0.001 (Dunnett’s test).

At 227 DAF, the contents of 15 carotenoid compounds in the flesh of the RB14 and W1 treatments were analyzed. Except for β-citraurin and 8′-apo-β-carotenal, light treatments significantly increased the contents of the other 13 carotenoids. Among xanthophylls, β-cryptoxanthin, lutein, violaxanthin, antheraxanthin, and zeaxanthin were the major carotenoids in satsuma mandarin flesh, with β-cryptoxanthin being the most abundant. RB14 and W1 increased the β-cryptoxanthin content by 120.3% and 97.6%, respectively, compared with the control, and this compound also showed the greatest absolute increase among all carotenoids. Phytoene was also relatively abundant in the flesh and was significantly induced by RB14 and W1 (Figure 6). Phytoene is the first colorless intermediate in plant carotenoid biosynthesis and plays a decisive role in carotenoid accumulation. The β-carotene content was also relatively high in satsuma mandarin flesh, suggesting that it may be a key carotenoid responsible for the red flesh color.

### 2.6. Supplementary Light Upregulates the Expression of Carotenoid Biosynthesis Genes in Flesh

**Figure 7:**
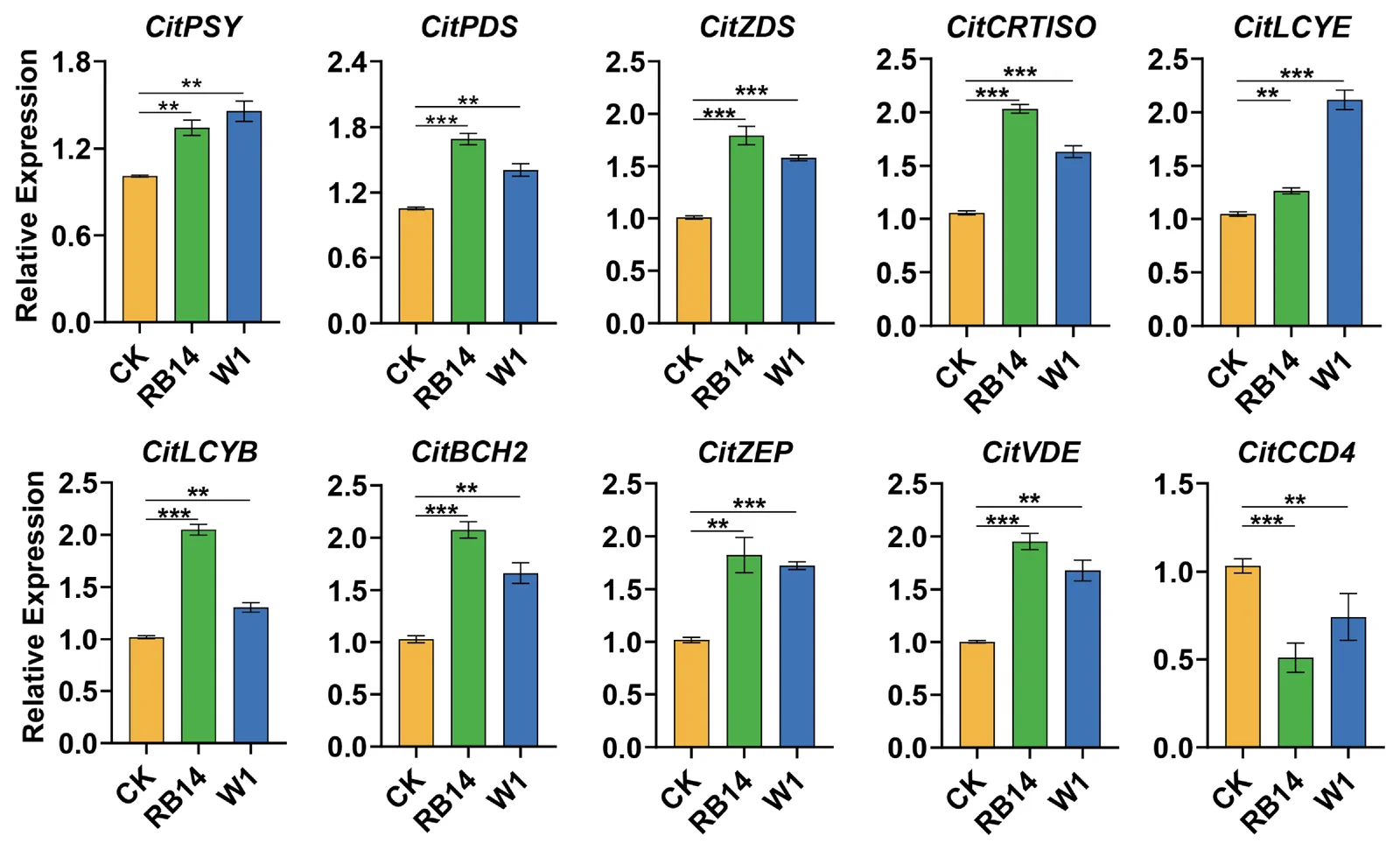
Expression of carotenogenic genes in the flesh under RB14 and W1 treatments. ** *p* < 0.01; and *** *p* < 0.001 (Dunnett’s test).

To investigate the molecular mechanisms underlying the supplementary light-induced carotenoid accumulation in fruit, the expression of nine key carotenoid biosynthesis genes and one carotenoid degradation gene, *CitCCD4*, was analyzed in the flesh. Among the nine biosynthetic genes, *CitPSY* encodes the rate-limiting enzyme that catalyzes the conversion of GGPP to phytoene, the first carotenoid intermediate. *CitPDS*, *CitZDS*, and *CitCRTISO* are involved in the conversion of phytoene to red lycopene; *CitLCYE* and *CitLCYB* control the synthesis of carotenes and xanthophylls; and *CitBCH2*, *CitZEP*, and *CitVDE* are responsible for the synthesis of β-cryptoxanthin, zeaxanthin, antheraxanthin, and violaxanthin. Supplementary light significantly upregulated all nine carotenoid biosynthesis genes, including the rate-limiting gene *CitPSY*, while significantly downregulating *CitCCD4* expression (Figure 7). These results indicate that the combined upregulation of carotenoid biosynthesis genes and downregulation of the degradation gene contributed to carotenoid accumulation in fruit under supplementary light.

## 3. Discussion

Light is an essential factor for plant growth and development, serving both as the energy source for photosynthetic carbon assimilation and as a regulator of plant physiological activities through changes in the photoperiod [33]. In this study, supplementary lighting was applied during winter when natural light intensity and daylength were reduced, aiming to further improve the nutritional quality of satsuma mandarin fruit, particularly the carotenoid content, and thereby increase economic benefits. After supplementary lighting, the fruit CCI increased significantly, and carotenoid accumulation in both the peel and the flesh was markedly enhanced. Meanwhile, the contents of sugars, organic acids, ascorbic acid, amino acids, flavonoids, and phenolic acids also increased significantly. In addition, supplementary lighting increased the leaf chlorophyll content and delayed its degradation, and the expression of genes involved in carotenoid biosynthesis was significantly upregulated.

Our results showed that supplementary lighting with different light qualities and different intensities of white light significantly promoted fruit coloration and increased the carotenoid content in satsuma mandarin. Light is a key factor stimulating carotenoid biosynthesis and accumulation in plants [34]. Artificial red and blue light irradiation significantly increased the total carotenoid and lycopene contents in tomato fruit [35]. Blue light enhanced the accumulation of phytoene, phytofluene, lycopene, and *cis*-lycopene in grapefruit peel, whereas red light increased the total carotenoid content, including β-carotene, in the peel [36]. Zhang et al. [16] also demonstrated that blue light at different intensities significantly increased the total carotenoid content in the juice sacs of satsuma mandarin, consistent with our findings. In addition, our results showed that β-cryptoxanthin was the most abundant carotenoid in satsuma mandarin fruit, and the RB14 and W1 treatments significantly increased its content. Ma et al. [15,37] also reported that red LED light significantly increased the β-cryptoxanthin content in satsuma mandarin fruit, and blue light at different intensities increased the β-cryptoxanthin content in juice sacs [16]. Taken together, these findings indicate that supplementary lighting is an effective approach to increase the carotenoid content in the peel and flesh of satsuma mandarin, particularly β-cryptoxanthin.

In addition to carotenoids, the contents of soluble sugars, ascorbic acid, total amino acids, flavonoids, and phenolic acids in satsuma mandarin fruit were also significantly increased under most supplementary lighting. Zhan et al. [14] reported that light treatment significantly increased the soluble sugar and ascorbic acid contents in fresh-cut celery during storage and extended its shelf life. During plant growth, mixed red and blue light treatment (R1B0.8) significantly enhanced sugar accumulation in tomato fruit and improved fruit quality [35]. Conversely, shading reduced the ascorbic acid accumulation in the flavedo of Navelina orange and Clementine mandarin [10]. Wilawan et al. [38] found that white and blue light treatments significantly increased the phenolic content in okra during growth, while red light treatment during postharvest storage of tomato significantly increased the phenolic and flavonoid contents [39]; blue light also increased total amino acid accumulation in fruit. These results are consistent with previous reports, demonstrating that supplementary lighting can significantly improve satsuma mandarin fruit quality.

Light is the energy source for photosynthesis, and chlorophyll is essential for capturing light and converting it into chemical energy for carbon assimilation. Our results showed that different ratios of red and blue light and different intensities of white light significantly increased the total chlorophyll content in satsuma mandarin leaves, delayed chlorophyll degradation, and increased the chlorophyll a/b ratio. Several studies have shown that the chlorophyll content and photosynthetic capacity decline under shading or reduced light intensity [40]. For example, the chlorophyll content in *Arabidopsis* leaves gradually decreased with decreasing light intensity [41], and in purple pak-choi (*Brassica campestris* ssp. *chinensis* Makino), chlorophyll and carotenoid accumulation, as well as photosynthetic capacity, decreased progressively under reduced light intensity. Light quality also strongly affects chlorophyll accumulation; blue light promoted chlorophyll accumulation and enhanced photosynthetic efficiency in *Dysosma versipellis* [42], and Wang et al. [43] reported that both red and blue light significantly increased the chlorophyll a/b ratio in grape leaves, with red light also significantly increasing the total chlorophyll content. Supplementation with mixed red and blue light significantly increased the chlorophyll content and enhanced photosynthetic capacity in tomato leaves, thereby significantly increasing the carotenoid content in the fruit and total biomass [20]. Therefore, based on these reports, we speculate that supplementary lighting increased the chlorophyll content in satsuma mandarin leaves, thereby promoting the accumulation of photosynthetic products and consequently enhancing the accumulation of nutrients such as carotenoids and sugars.

The precursor for plant carotenoid biosynthesis is GGPP, a product of photosynthesis that is subsequently converted into various carotenoids through a series of enzymatic reactions. Therefore, the expression levels of carotenoid biosynthesis genes determine carotenoid accumulation. Phytoene synthase (PSY) is the key rate-limiting enzyme that catalyzes the conversion of GGPP to phytoene, the first carotenoid [5], and thus, *PSY* expression plays a decisive role in determining the carotenoid content in plants [44]. In this study, both the RB14 and W1 treatments significantly upregulated *CitPSY* expression in satsuma mandarin fruit, leading to a significant increase in phytoene and other carotenoid contents in the flesh. Phytoene is subsequently converted into colored carotenoids through the action of downstream carotenoid biosynthesis genes [5]. Zhang et al. [16] reported that blue light upregulated the expression of *CitPSY*, *CitPDS*, *CitZDS*, *CitLCYb2*, and *CitHYb* in the juice sacs of satsuma mandarin and navel orange, resulting in significant increases in total carotenoids, α-carotene, β-carotene, β-cryptoxanthin, and lutein. Conversely, shading of Clementine mandarin and sweet orange fruit downregulated the expression of carotenoid biosynthesis genes such as *PSY*, *PDS*, *ZDS1*, *LCY2a*, and *LCY2b* in the peel [11]. Gong et al. [36] reported that blue or red light irradiation during postharvest storage of ‘Star Ruby’ grapefruit significantly upregulated the expression of *PSY*, *PDS*, *ZDS*, *CRTISO*, *LCYb2*, *BCH2*, and *BCH3* in the peel and increased the peel carotenoid content, and similar results were obtained in satsuma mandarin under red light during postharvest [37]. These findings indicate that the upregulation of key carotenoid biosynthesis genes by RB14 and W1 supplementary light is an important factor contributing to increased carotenoid accumulation in the flesh. Furthermore, *CitCCD4* is a key gene encoding the enzyme responsible for β-citraurin synthesis in citrus; the CitCCD4 enzyme cleaves β-cryptoxanthin and zeaxanthin to form β-citraurin [45,46]. In this study, RB14 and W1 significantly downregulated *CitCCD4* expression in the flesh, while the β-citraurin content did not differ significantly from the control. In addition, *CitBCH2* expression was significantly upregulated. Together, these changes contributed to the substantial accumulation and significant increase in β-cryptoxanthin content in the flesh.

## 4. Materials and Methods

### 4.1. Plant Materials

Twelve-year-old satsuma mandarin (*Citrus unshiu* Marc. cv. Miyagawa) trees, grafted onto trifoliate orange (*Citrus trifoliata*) rootstocks and cultivated under greenhouse conditions in Xinggan County, Jiangxi Province, China, were selected for uniformity in growth vigor and fruit load. The experimental area had red soil with a pH of 4.6, an average annual temperature of 17.5 °C, and an average annual rainfall of 1604.5 mm. From September to November, the air temperature in the experimental greenhouse ranged from 15 to 30 °C, and the relative humidity ranged from 75% to 85%. After November, the air temperature ranged from 5 to 15 °C, and the relative humidity ranged from 65% to 80%.

### 4.2. Supplementary Light Treatments

Supplementary lighting was initiated on 23 September (156 days after flowering, DAF). From this date, when the daily photoperiod was shorter than 12 h, until 7 November, 2 h of supplementary light was provided before sunrise and 2 h after sunset each day, giving a total of 4 h per day. After 7 November, when the photoperiod gradually decreased below 10 h, 3 h of supplementary light was provided before sunrise and 3 h after sunset each day until fruit harvest, giving a total of 6 h per day.

White reflective film was laid on the ground under both the control and light-treated trees. Light treatments included red light (660 nm), blue light (470 nm), mixed red and blue light, and white light of different intensities. The photosynthetic photon flux density (PPFD) was measured using a LightScout Quantum Light Meter 3415F (Spectrum Technologies, Inc., Aurora, IL, USA). Each treatment had three biological replicates. Each biological replicate consisted of five trees, and each treatment therefore comprised 15 trees. Two trees were left as a buffer between adjacent treatments to prevent light spillover. All LED supplementary lamps were custom-made by Xiamen Lumigro Technology Co., Ltd. (Xiamen, China) according to the experimental requirements. The detailed treatment scheme is shown in Table 5. Following the light treatments, fruit and leaf samples were collected at 193 DAF, 212 DAF, 227 DAF, 241 DAF, and 250 DAF for subsequent analyses.

### 4.3. Peel Color Contribution Index Analysis

Peel color was measured using a CR-400 handheld colorimeter (Konica Minolta, Tokyo, Japan) with a D65 light source, based on the CIELab color space. The *L*, *a*, and b* values were recorded at three evenly distributed points around the equatorial plane of each fruit. Nine fruits were measured per replicate, with three biological replicates. The L* value represents lightness (higher values indicate greater brightness), a* represents the red–green axis (positive values indicate redness, negative values greenness), and b* represents the yellow–blue axis (positive values indicate yellowness, negative values blueness). The color contribution index (CCI) was calculated as CCI = 1000 × a*/(L* × b*).

### 4.4. Determination of Chlorophyll and Total Carotenoid Contents

Fresh leaves (0.2 g, midrib removed), peel (0.1 g, albedo removed), or flesh (0.5 g) samples were cut into small pieces, homogenized, and ground; then 8 mL of 80% acetone was added. After standing for 3–5 min, the mixture was centrifuged at 1100× *g* for 10 min. The supernatant was transferred to a new tube, and the absorbance was measured at 663 nm, 646 nm, and 470 nm. The chlorophyll and total carotenoid contents were calculated according to the following formulas:

*C_a_* (μg/g) = (12.21*A*_663_ − 2.81*A*_646_) × 8/m (1)

*C_b_* (μg/g) = (20.13*A*_646_ − 5.03*A*_663_) × 8/m (2)

*C_T_* = *C_a_* + *C_b_* (3)

*C_X.C_* (μg/g) = (1000*A*_470_ − 3.27*C_a_* − 104*C_b_*) × 8/(229 × m) (4)

The chlorophyll a (*C_a_*), chlorophyll b (*C_b_*), total chlorophyll (*C_T_*), and total carotenoid (*C_X.C_*) contents were calculated based on the measured absorbance values and the sample fresh weight (m).

### 4.5. Determination of Fruit Quality

After fruit collection, the juice was extracted using a juicer. The total soluble solids (TSSs) content was measured using a PAL-1 digital handheld refractometer (ATAGO Co., Ltd., Tokyo, Japan). The titratable acid content was determined by NaOH titration. The soluble sugar content was measured using the anthrone colorimetric method. The vitamin C (ascorbic acid) content was determined by the 2,6-dichloroindophenol titration method [31].

### 4.6. Determination of Total Amino Acids, Flavonoids, and Phenolics

Fruit samples from the RB14 and W1 treatments were collected at 193 DAF, 212 DAF, 227 DAF, 241 DAF, and 250 DAF. The total amino acid content in the peel and flesh was determined using a Total Amino Acid (T-AA) Assay Kit (colorimetric method) (Nanjing Jiancheng Bioengineering Institute Co., Ltd., Nanjing, China, Product Number: A026-1-1) according to the manufacturer’s instructions (http://www.njjcbio.com/products.asp?id=332 (accessed on 1 October 2026)). Briefly, a standard curve was prepared using glycine standards. Peel or flesh samples (0.1 g) were accurately weighed, homogenized in physiological saline at a ratio of 0.1 g:0.9 mL, and centrifuged at 4000 rpm for 10 min. Then, 0.5 mL of the supernatant was mixed with 0.5 mL of Reagent 5, vortexed, and centrifuged at 4000 rpm for 10 min. The supernatant was collected, and the amino acid reaction solution and chromogenic reagent were added for color development. The absorbance was measured at 650 nm, and the amino acid concentration was calculated based on the standard curve.

The total phenolic content (TPC) was quantified using the Folin–Ciocalteu assay. Gallic acid was used as a standard to calculate the phenolic content, and the data were expressed as milligrams of gallic acid equivalent per 100 g fresh weight (mg GAE/100 g FW). The total flavonoid content (TFC) was determined by the sodium borohydride/chloranil (SBC) assay. Catechin was used as a standard to calculate the flavonoid content, and the data were expressed as milligrams of catechin equivalent per 100 g fresh weight (mg CE/100 g FW) [47].

### 4.7. Identification and Quantification of Carotenoid Species

Fruit samples from the RB14 and W1 treatments were collected at 227 DAF. The identification and quantification of carotenoid species were performed following the method of Kato et al. [24]. Fresh flesh (3 g) was weighed, homogenized, and placed in a 50-mL centrifuge tube. Then, 15 mL of carotenoid extraction solution was added, and the mixture was vortexed. The mixture was vigorously shaken in a grinding mill for 6 min and then centrifuged at 4000× *g* for 10 min. The supernatant was transferred to another 50 mL centrifuge tube (a standard centrifuge tube was sufficient). The pellet was re-extracted twice with 15 mL of the same extraction solution until colorless (three extractions in total), and the supernatants were combined in the same centrifuge tube. The combined supernatant was washed three times with 10% (*w*/*v*) NaCl aqueous solution in a 50 mL separatory funnel until neutral, and the lower aqueous layer was discarded. The upper phase was transferred in portions to 10 mL centrifuge tubes and concentrated to dryness under vacuum. The residue was dissolved in 2 mL of Methyl t-Butyl Ether (MTBE) containing 0.01% (*w*/*v*) 2,6-Di-bert-buty-4-methyl-phenol (BHT), and 2 mL of 10% (*w*/*v*) KOH in methanol containing 0.01% (*w*/*v*) BHT was added. The mixture was saponified in the dark for 10 h. Then, 4 mL of 10% (*w*/*v*) NaCl aqueous solution and 2 mL of MTBE containing 0.01% (*w*/*v*) BHT were added to improve phase separation. The aqueous layer was removed with a Pasteur pipette, and the organic phase was washed three times with 5 mL of 10% (*w*/*v*) NaCl aqueous solution until neutral. The remaining pigment-containing supernatant was transferred in portions to 2 mL centrifuge tubes and concentrated to dryness under vacuum. The dried extract could be stored temporarily at −80 °C and redissolved before analysis. The residue was then dissolved and made up to a final volume of 1 mL with MTBE. The solution was centrifuged at 15,871× *g* for 30 min, filtered through a membrane filter, transferred to a sample vial, and analyzed by high-performance liquid chromatography (HPLC). HPLC analysis was performed using a Waters (Waters, Milford, MA, USA) equipped with a 1525 HPLC pump system, a 717 autosampler, a 2996 photodiode array detector, and Empower 2 chromatography data software, with a reversed-phase C30 carotenoid column (250 × 4.6 mm long column/150 × 4.6 mm short column; YMC Co., Ltd., Kyoto, Japan).

HPLC mobile phase preparation: A, acetonitrile:methanol = 3:1 (750:250 mL, containing 0.01% BHT); B, 100% MTBE (containing 0.01% BHT). After preparation, each mobile phase was filtered under vacuum through a 0.22 μm organic membrane filter to remove impurities. The mobile phases were then sonicated in an ultrasonic bath for 30 min to degas them.

### 4.8. RNA Extraction and Gene Expression Analysis

Total RNA was extracted from the flesh of satsuma mandarin fruits treated with RB14 and W1 at 227 DAF using the Trizol method [48]. Fresh flesh samples were placed in a pre-cooled grinding jar, ground to a fine powder in liquid nitrogen using a Mixer Mill MM 400 (Retsch GmbH, Haan, Nordrhein-Westfalen, Germany), and 0.9 g of the ground tissue was mixed vigorously with TRIzol Buffer, followed by incubation at room temperature for 15 min. The RNA was then extracted with chloroform and isopropanol. First-strand cDNA was synthesized from the extracted RNA using the PrimeScript™ RT reagent Kit with gDNA Eraser (Code No. RR047A, Takara Biomedical Technology Co., Ltd., Beijing, China) and used for quantitative real-time PCR (qRT-PCR).

Primers for qRT-PCR were designed using Primer 5.0 software. The actin gene was used as an internal reference, and the primer sequences for all genes are listed in Supplementary Table S2. Gene expression analysis was performed on a Bio-Rad CFX96 Real-Time PCR System (Bio-Rad Laboratories, Hercules, CA, USA) using SYBR Green master mix. Each 20 μL reaction contained 10 μL of 2× SYBR Green master mix, 0.4 μL of each primer (10 μM), 2 μL of diluted cDNA, and 7.2 μL of nuclease-free water. The thermal cycling program consisted of an initial denaturation at 95 °C for 3 min, followed by 40 cycles of 95 °C for 10 s and 60 °C for 30 s. A melt curve was generated from 60 °C to 95 °C at a ramp rate of 0.2 °C/s to verify primer specificity. Primer amplification efficiencies were determined using a 10-fold dilution series of pooled cDNA, and only primers with efficiencies between 85% and 110% (R^2^ > 0.98) were used. The Actin gene was used as an internal reference, and its expression stability was confirmed by a coefficient of variation < 5% across all samples. Relative gene expression was calculated using the 2^−ΔΔCt^ method. Three biological replicates and three technical replicates were performed for each sample.

### 4.9. Statistical Analysis

All data are presented as the mean ± standard error (SE). Significant differences among treatment means were determined by two-way ANOVA (SPSS 23.0) followed by Tukey’s HSD multiple range test or Dunnett’s *t*-test. Figures were generated using GraphPad Prism 10 and Adobe Illustrator 2022.

## 5. Conclusions

**Figure 8:**
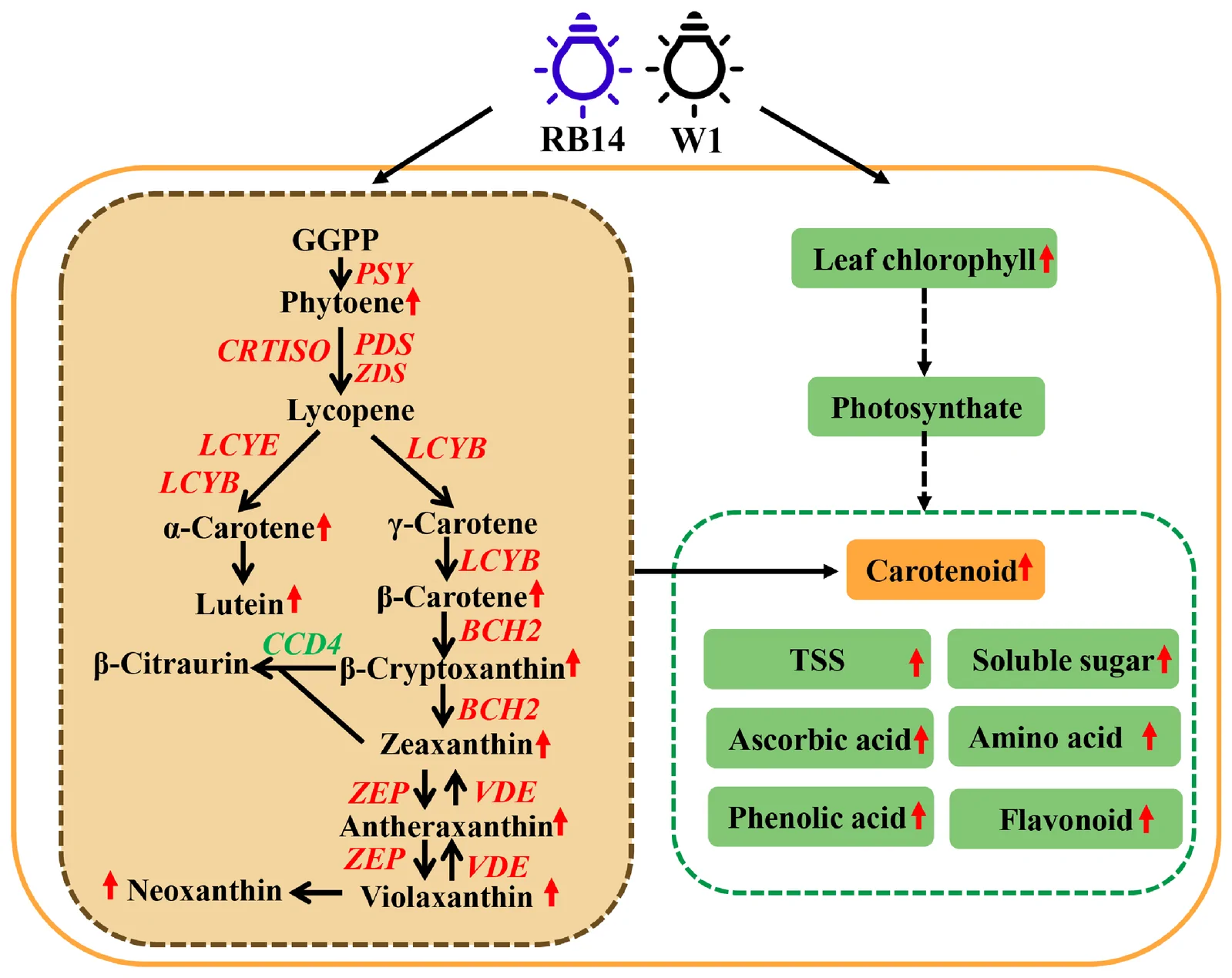
Schematic diagram illustrating the potential physiological and molecular mechanisms by which RB14 and W1 supplementary light treatments increase carotenoid content and other quality attributes in satsuma mandarin fruit. Gene names in red indicate upregulated expression, gene names in green indicate downregulated expression, red arrows indicate increased substance content.

This study investigated the effects of supplementary red–blue light and white light on the carotenoid content and related fruit quality traits of satsuma mandarin under reduced natural light intensity and shorter day length conditions in autumn and winter. We also analyzed the effects of supplementary light on the leaf chlorophyll content and the expression of key carotenoid biosynthesis genes in the flesh. The results showed that all supplementary light treatments increased total carotenoid accumulation in both the rind and flesh; improved other fruit quality attributes such as sugars, ascorbic acid, and amino acids; and significantly increased the leaf chlorophyll content while delaying its degradation. Among the treatments, RB14 and W1 exhibited the strongest effects. These findings suggest that supplementary lighting is associated with increased carotenoid and other nutrient accumulation in fruit, possibly through increased leaf chlorophyll content and enhanced accumulation of photosynthetic products. Furthermore, the RB14 and W1 treatments upregulated the expression of key carotenoid biosynthesis genes in the flesh, including *CitPSY*, *CitPDS*, *CitZDS*, *CitCRTISO*, *CitLCYE*, *CitLCYB*, *CitBCH2*, *CitZEP*, and *CitVDE*, and downregulated *CitCCD4* expression; these changes may contribute to carotenoid accumulation (Figure 8). In conclusion, supplementary red–blue light or white light may represent an effective approach to improve the nutritional value of satsuma mandarin fruit.

## Figures and Tables

**Table 1:** Total soluble solids (TSSs) content in fruit under different supplementary light treatments.

DAF	Total Soluble Solid (%)							
	CK	R	B	RB11	RB14	RB41	W1	W2
193	11.67 ± 0.37 ab	12.60 ± 0.23 ab	12.37 ± 0.21 ab	11.40 ± 0.19 b	12.13 ± 0.21 ab	12.53 ± 0.30 ab	12.00 ± 0.14 ab	12.77 ± 0.11 a
212	11.83 ± 0.73 a	13.77 ± 0.26 a	13.00 ± 0.34 a	13.17 ± 0.31 a	13.23 ± 0.88 a	13.10 ± 0.50 a	13.47 ± 0.26 a	12.20 ± 0.05 a
227	11.15 ± 0.38 c	13.86 ± 0.35 ab	14.10 ± 0.57 a	14.44 ± 0.27 a	14.10 ± 0.66 a	12.32 ± 0.14 bc	13.76 ± 0.34 ab	14.00 ± 0.28 a
241	12.40 ± 0.24 c	14.34 ± 0.46 ab	13.80 ± 0.10 abc	13.58 ± 0.30 abc	14.73 ± 0.31 a	12.94 ± 0.26 bc	14.70 ± 0.57 a	14.08 ± 0.52 ab
250	12.20 ± 0.06 c	14.33 ± 0.07 ab	14.43 ± 0.39 ab	13.30 ± 0.31 bc	15.65 ± 0.29 a	13.50 ± 0.45 bc	14.45 ± 0.23 ab	13.27 ± 0.17 bc

Note: All values are shown as the means ± SE. The data were analyzed, and Tukey’s HSD test was used to compare the means at *p* < 0.05, Different lowercase letters following the data indicate significant differences among supplementary light treatments at the same time point.

**Table 2:** Soluble sugar content in fruit under different supplementary light treatments.

DAF	Soluble Sugar (%)							
	CK	R	B	RB11	RB14	RB41	W1	W2
193	9.96 ± 1.01 a	11.99 ± 0.30 a	11.32 ± 1.06 a	8.70 ± 0.30 a	11.99 ± 0.52 a	11.54 ± 1.30 a	10.86 ± 0.99 a	10.13 ± 0.44 a
212	8.25 ± 0.38 b	9.48 ± 0.43 ab	9.67 ± 0.40 ab	9.29 ± 0.13 ab	10.39 ± 0.50 a	9.62 ± 0.57 ab	10.15 ± 0.19 a	10.42 ± 0.81 a
227	9.21 ± 0.36 b	10.56 ± 0.57 ab	10.98 ± 0.38 ab	10.56 ± 0.48 ab	11.87 ± 0.32 a	10.72 ± 0.10 ab	11.38 ± 0.33 a	11.50 ± 0.35 a
241	9.99 ± 0.57 b	12.41 ± 0.11 a	12.37 ± 0.19 a	11.51 ± 0.51 ab	11.45 ± 0.21 ab	10.88 ± 0.18 ab	11.56 ± 0.30 ab	11.72 ± 0.48 ab
250	11.32 ± 0.33 a	13.22 ± 0.11 a	12.85 ± 0.90 a	11.67 ± 0.67 a	11.15 ± 1.13 a	10.27 ± 0.99 a	11.25 ± 0.97 a	11.36 ± 0.60 a

Note: All values are shown as the means ± SE. The data were analyzed, and Tukey’s HSD test was used to compare the means at *p* < 0.05, Different lowercase letters following the data indicate significant differences among supplementary light treatments at the same time point.

**Table 3:** Titratable acid content in fruit under different supplementary light treatments.

DAF	Titratable Acid (%)							
	CK	R	B	RB11	RB14	RB41	W1	W2
193	0.73 ± 0.05 ab	0.69 ± 0.02 ab	0.85 ± 0.04 a	0.73 ± 0.02 ab	0.67 ± 0.05 ab	0.55 ± 0.08 b	0.73 ± 0.08 ab	0.69 ± 0.04 ab
212	0.54 ± 0.02 a	0.60 ± 0.02 a	0.67 ± 0.04 a	0.64 ± 0.05 a	0.66 ± 0.08 a	0.63 ± 0.06 a	0.62 ± 0.08 a	0.68 ± 0.06 a
227	0.42 ± 0.02 a	0.41 ± 0.01 a	0.42 ± 0.01 a	0.44 ± 0.02 a	0.42 ± 0.02 a	0.30 ± 0.02 b	0.44 ± 0.02 a	0.42 ± 0.02 a
241	0.47 ± 0.02 abc	0.54 ± 0.03 ab	0.49 ± 0.03 abc	0.49 ± 0.03 abc	0.58 ± 0.02 a	0.45 ± 0.02 bc	0.41 ± 0.02 c	0.47 ± 0.02 abc
250	0.48 ± 0.02 a	0.44 ± 0.03 a	0.46 ± 0.04 a	0.48 ± 0.02 a	0.48 ± 0.02 a	0.40 ± 0.02 a	0.46 ± 0.02 a	0.39 ± 0.03 a

Note: All values are shown as the means ± SE. The data were analyzed, and Tukey’s HSD test was used to compare the means at *p* < 0.05, Different lowercase letters following the data indicate significant differences among supplementary light treatments at the same time point.

**Table 4:** Ascorbic acid content in fruit under different supplementary light treatments.

DAF	Ascorbic Acid (mg/100 g)							
	CK	R	B	RB11	RB14	RB41	W1	W2
193	19.83 ± 0.78 c	16.11 ± 0.78 d	17.94 ± 0.40 cd	21.00 ± 0.55 bc	21.45 ± 0.46 bc	25.62 ± 0.76 a	23.43 ± 0.70 ab	21.09 ± 0.59 bc
212	15.08 ± 0.19 bc	17.88 ± 0.77 abc	18.74 ± 0.67 ab	18.96 ± 0.69 a	13.79 ± 0.51 d	18.74 ± 0.67 ab	15.15 ± 0.83 bcd	15.73 ± 0.77 abcd
227	19.62 ± 0.76 d	23.45 ± 0.28 c	29.25 ± 0.28 a	25.06 ± 0.50 bc	26.03 ± 0.50 abc	28.29 ± 0.76 ab	25.70 ± 1.14 abc	27.98 ± 0.88 ab
241	24.82 ± 1.01 b	27.59 ± 0.78 ab	26.62 ± 0.75 ab	27.88 ± 0.50 ab	28.21 ± 0.76 ab	25.96 ± 0.86 ab	29.17 ± 0.57 a	28.22 ± 0.76 ab
250	23.08 ± 0.79 c	26.28 ± 0.64 bc	25.32 ± 0.64 bc	29.81 ± 0.79 ab	32.69 ± 0.96 a	30.23 ± 1.13 ab	30.77 ± 0.79 ab	27.88 ± 1.47 abc

Note: All values are shown as the means ± SE. The data were analyzed, and Tukey’s HSD test was used to compare the means at *p* < 0.05, Different lowercase letters following the data indicate significant differences among supplementary light treatments at the same time point.

**Table 5:** Experimental design of supplementary light treatments.

Treatment	Photon Flux Density ^a^ (μmol m^−2^s^−1^)
CK	0
R	130
B	130
RB11	65 (Red) + 65 (Blue)
RB14	26 (Red) + 104 (Blue)
RB41	104 (Red) + 26 (Blue)
W1	350
W2	510

^a^: all photon flux densities refer to those measured at the top of the canopy. The LED supplementary lamps were positioned 50 cm above the top of the tree canopy.

## Data Availability

The original contributions presented in this study are included in the article. Further inquiries can be directed to the corresponding author.

## Supplementary Materials

The following supporting information can be downloaded at: https://www.mdpi.com/article/10.3390/plants15193067/s1, Table S1: Chlorophyll a and chlorophyll b contents in leaves under different supplementary light treatments; Table S2: Primers used for expression analysis of carotenogenic genes.
